# Supplementary material for: Invasion Pattern of Aedes aegypti in the Native Range of Ae. albopictus in Vietnam Revealed by Biogeographic and Population Genetic Analysis
Source: Insects. 2022 Nov 23;13(12):1079. doi: 10.3390/insects13121079 (PMC9782358; doi:10.3390/insects13121079)
Supplement: Supplementary file 1 [file insects-13-01079-s001.zip › insects-1933050-Supplementary.pdf]

Supplementary

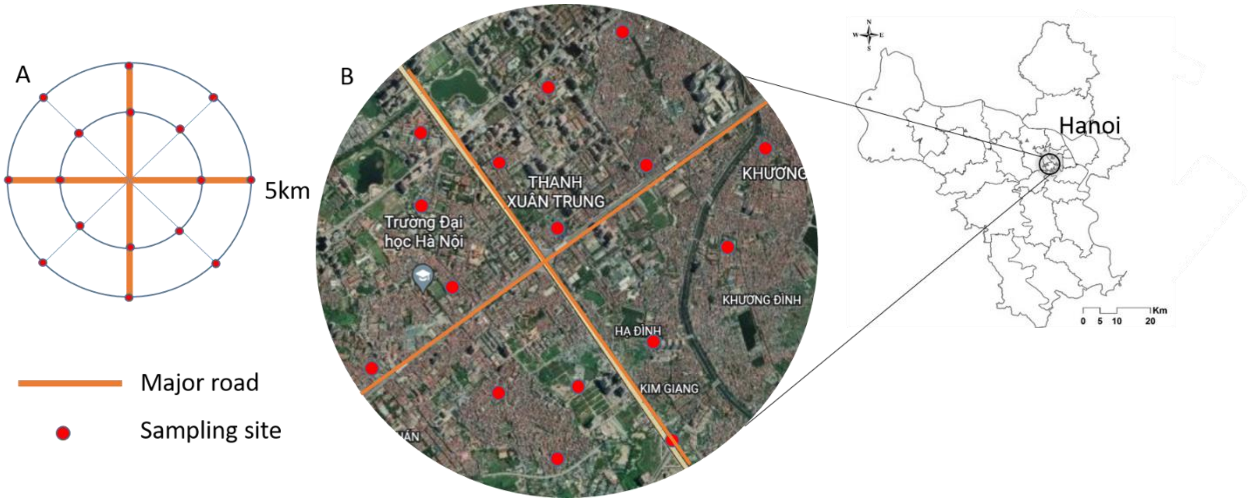

Figure S1. Sampling design for *Aedes* mosquitoes in a study city. A. Sampling scheme for each study site; B. An example of sampling design with selected sampling points in urban areas of a study site (S7-Hanoi city), map was retrieved from Google earth V 9.172.0.0, http://www.earth.google.com.

Table S1. Effects of species, climate, habitat, and their interactions on *Aedes aegypti* and *Ae. albopictus* abundance (Multi-Way ANOVA)

No.	Model	DFn	DFd	*F*-statistic	*P*-value
1	Species	1	102	6.253	0.014
2	Region	1	102	1.615	0.27
3	Habitat	2	102	98.786	<0.00001
4	Species:Region	1	102	81.021	<0.00001
5	Species:Habitat	2	102	17.434	<0.00001
6	Region:Habitat	2	102	1.047	0.355
7	Species:Region:Habitat	2	102	41.994	<0.00001

Table S2. Partitioning of genetic variation among *Aedes aegypti* populations in Vietnam

Hypotheses	Source of variation	Percentage of variation	F-statistic	*P* value
1. Two groups: North and Centre + South<br>- North (S07, S08, S09)<br>- Centre + South (S12, S14, S15, S17, S18)	Φ_{CT}	4.42	0.0442	0.02
	Φ_{SC}	-2.30	-0.0241	0.78
	Φ_{ST}	97.88	0.0211	0.43
2. Three groups: North, Central and South<br>- North (S07, S08, S09)<br>- Central (S12, S14, S15)<br>- South (S17, S18)	Φ_{CT}	2.33	0.0234	0.113
	Φ_{SC}	-1.70	-0.0174	0.652
	Φ_{ST}	99.36	0.0064	0.433

Table S3. Geographic origin and *COI* sequences of *Aedes aegypti* used in phylogeographic analysis. COI sequences were retrieved from GenBank (https://www.ncbi.nlm.nih.gov/nuccore/; Accessed on 23 September 2021)

Geographical area	Country	Accession Number
Central Africa	Cameroon	JQ926702
West Africa	Bennin	KX446400
	Bennin	KX446402
	Guinea	HQ688292
	Guinea	HQ688293
East Africa	Kenya	KX420438
	Kenya	KX420460
	Kenya	MK533627
	Uganda	KX446420
	Uganda	KX446438
North America	Mexico	MT108569
	Mexico	MT108574
	Mexico	MT108661
	USA	GU907840
	USA	GU907841
	USA	JQ926684
Caribbean	Martinique	JQ926696
	Venezuela	JQ926701
Central America	Panama	KX171389
	Panama	KX171390
	Panama	KX171394
South America	Brazil	JQ926703
	Chile	HQ991718
	France: Guyana	HQ688297
	Peru	MN299016
	Argentina	MN299024
Indian Ocean	France: Europa Island	HQ688296
Southwest Asia	India	MK542379
	India	MK635053
	Pakistan	KF406349
Southeast Asia	Thailand	HQ688295
	Thailand	JQ926691
	Thailand	JQ926692
	Thailand	KM613046
	Thailand	KM613048
	Thailand	KM613055
	Cambodia	HQ688294
	Cambodia	JQ926688
	Cambodia	JQ926689
	Cambodia	JQ926690
	Malaysia	MF148257
	Malaysia	MF148262
	Vietnam	JQ926686, 87

Table S4. Worldwide survey of haplotype similarities of Vietnamese *Aedes aegypti* haplotypes. COI sequences were retrieved from GenBank (https://www.ncbi.nlm.nih.gov/nuccore/; Accessed on 23 September 2021).

Haplotype	GenBank code	Region	Country
H1	MN299016	South America	Peru
	MN299008	South America	Puerto Rico
	MK542380	South Asia	India
	KC970271	South Asia	India
	MF443395-97	North America	Canada
	MF148262, MF148257	Southeast Asia	Malaysia: Selangor
	MF043259	Europe	United Kingdom: England
	KY022526	Europe	Germany
	MW961318, 19	Europe	Portugal
	MZ230361	Southeast Asia	Vietnam
	MK505603, MK505600, 01 MK505698, MK505695, MK505685,86 MK505679, MK505677, MK505672	Europe	United Kingdom: England
	MW509605	South America	Puerto Rico
	MT108574	North America	Mexico
	MN733743, MN733745-53	Pacific Ocean	New Caledonia
	HQ991718	South America	Chile
	AF425846	North America	Canada, Lab conlony
H2	MZ230363	Southeast Asia	Vietnam
	GU675484	Pacific Ocean	Australia
H3	MK890402	South America	Ecuador
	KX420476	South America	Ecuador
	MZ230362	Southeast Asia	Vietnam
	MT108569	North America	Mexico
	AY432106, AY432648	Europe	United Kingdom: Liverpool-strain
H7	KF406376	Southwest Asia	Pakistan
	HM399357	Pacific Ocean	Australia
	MZ230364	Southeast Asia	Vietnam

Table S5. Correlation between genetic diversity and abundance of *Ae. aegypti* and *Ae. albopictus* across Vietnam.

Co-occurence	Region	*Ae. aegypti*		*Ae. albopictus*	
		Hd, π	No. of individual/site	Hd, π*	No. of individual/site
No	Northeast (S4, S5, S6)	NC	NC	0.760, 0.0022	432±43.6
	Northwest (S1, S2, S3)			0.9016, 0.0034	1112±238.2
Yes	North Delta and North Central coast (S7, S8, S9, S10, S11)	0.8413, 0.0100	202.8±156.5	0.8763, 0.0032	878±91.8
	Southern (S12, S13, S14, S15, S16, S17, S18)	0.8882, 0.0091	868±276.5	0.8308, 0.0025	401±143.1

Hd: Haplotype diversity, π: nucleotide diversity, NC: not computed, *genetic diversity indices data of *Aedes albopictus* were retrieved from Duong et al. 2021[52].
